# Comparative Effects of Neurodynamic Slider and Tensioner Mobilization Techniques on Sympathetic Nervous System Function: A Randomized Controlled Trial

**DOI:** 10.3390/jcm13175098

**Published:** 2024-08-28

**Authors:** Charalambos Papacharalambous, Christos Savva, Christos Karagiannis, Eleftherios Paraskevopoulos, George M. Pamboris

**Affiliations:** 1 Department of Health Sciences, School of Sciences, European University Cyprus, Nicosia 2404, Cyprus; c.karayiannis@euc.ac.cy (C.K.); g.pamboris@euc.ac.cy (G.M.P.); 2 Department of Life and Health Sciences, Frederick University, Limassol 3080, Cyprus; c.savva@frederick.ac.cy; 3 Department of Physiotherapy, University of West Attica, 122 43 Athens, Greece; elparaskevop@uniwa.gr

**Keywords:** neurodynamic technique, tensioner technique, slider technique, long sitting slump, sympathetic nervous system

## Abstract

**Objective**: To investigate the effect of slider and tensioner neurodynamic techniques (NDTs) on the sympathetic nervous system (SNS) activity, aiming to identify which technique more effectively modulates autonomic responses in asymptomatic individuals. **Materials and Methods**: In this double-blind controlled trial, a total of 90 healthy participants were randomly allocated into three groups: slider, tensioner, and control. Skin conductance (SC) was continuously monitored throughout the entire 20 min experiment, while body temperature and blood pressure were measured pre- and post-intervention. **Results**: The SC levels significantly increased in both the slider and tensioner groups compared to the control group during the intervention and end rest period on the left leg (slider vs. control: *p* < 0.001, d = 1.20; tensioner vs. control: *p* < 0.001, d = 1.64) and on the right leg (slider vs. control: *p* < 0.001, d = 1.47; tensioner vs. control: *p* < 0.001, d = 0.73). There were no significant differences between the two NDTs on the left (*p* < 0.13, d = 0.89) and right legs (*p* < 1.00, d = 0.36). The body temperature of the slider group showed a significant increase compared to both the control group (*p* < 0.001, d = 0.95) and the tensioner group (*p* < 0.001, d = 1.48). There were no significant differences between the groups in systolic (*p* = 0.95) or diastolic blood pressure (*p* = 0.06). There were no side-specific effects on SNS activity between the left and right legs (*p* < 0.019) during all intervention phases. **Conclusions**: Significant sympathoexcitatory responses were elicited by both slider and tensioner NDTs in asymptomatic participants, demonstrating their efficacy in modulating the SNS. The differences between the two techniques were not statistically significant; however, the tensioner NDT showed a slightly more pronounced effect, suggesting that the tensioner NDT can be considered superior in terms of overall SNS effect. These findings indicate that both techniques may have the potential to enhance autonomic regulation in clinical practice; however, the tensioner NDT may be more effective. The consistent responses across participants highlight the systemic benefits of NDTs, providing a foundation for further research into their application in symptomatic populations. This study contributes to evidence-based practice by providing baseline data that support the development of theoretical frameworks and aid in clinical decision-making.

## 1. Introduction

Neurodynamic techniques (NDTs) are manual therapy techniques that focus on enhancing the mechanical function and mobility of peripheral nerves [1,2]. This is achieved by optimizing movement and reducing nerve tension within the nervous system and surrounding tissues [3,4]). In clinical practice, there are two commonly used variants of NDTs. The first is tensioner NDT, which involves moving nerve endings in opposite directions, significantly increasing nerve strain [5]. The second is slider NDT, in which nerve endings move in the same direction, allowing nerve mobilization with minimal strain increments [5,6]. Applying tensile forces, a form of mechanical loading, is essential for maintaining homeostasis in the nervous system [6,7]. Both techniques involve specific neuromobilization maneuvers that stimulate the nervous system [1,8,9,10]. Research indicates that NDTs can affect intraneural circulation and axoplasmic flow, which may influence the conduction of nerve impulses [3,10,11].

NDTs improve nerve mobility and reduce muscle tension, potentially mitigating pain by facilitating nerve gliding [12,13], reducing intraneural pressure, and modulating pain-related nervous system responses, including those of the sympathetic nervous system (SNS) [9,14]. By modifying the SNS, NDTs can influence posture and muscle tension, improving muscle tone and alignment, which promotes balanced muscle action and reduces compensatory responses, ultimately affecting pain perception [9]. This improvement in posture and muscle action not only enhances physical function but also contributes to the patient’s overall well-being [15]. The autonomic nervous system (ANS) regulates involuntary physiological processes such as heart rate, digestion, and respiratory rate [16,17]. The ANS, which consists of the SNS responsible for the “fight-or-flight” response and the parasympathetic nervous system responsible for relaxation and restoration, plays a crucial role in regulating bodily processes [18,19,20]. The dysregulation of the ANS can significantly affect one’s overall well-being [21]. This dysregulation is often associated with conditions such as hypertension, arrhythmias, and digestive problems [15,22].

A comprehensive understanding of the ANS is essential in the field of physiotherapy [12]. Physiotherapists rehabilitating patients with various health conditions need to acknowledge the significance of the ANS in vital bodily functions such as the heart rate, which is essential for exercise and recovery [14,17,22]. Identifying and dealing with autonomic dysregulation is crucial for optimizing treatment results, especially in stress, pain management, and cardiovascular health cases.

Recent research on the SNS has gained significant attention [23,24]. Multiple studies have focused on verifying the clinical effectiveness of NDTs, specifically their impact on the regulation of SNS activity and related physiological reactions. These studies indicate that improving nerve mobility and reducing nerve tension may relieve pain, improve blood circulation [4,18], and potentially affect the role of the SNS in regulating muscle tone [25,26]. NDTs can have a beneficial effect on reducing sympathetic activity, particularly in situations where neural restrictions contribute to increased sympathetic responses [19,27]. Moreover, dorsal periaqueductal gray (dPAG) stimulation via NDTs can result in hypoalgesic and sympathoexcitatory responses [4,9,28]. These treatment effects on the spinal segment may have a broader influence, indicating a potential global effect on pain and sympathetic-activity regulation in the entire body [23].

The effect of NDTs on the SNS is intricate and not completely understood, with results differing depending on the specific NDT used [18]. Prior research indicates that different NDT factors, such as loading type, magnitude, duration, rate of loading, and frequencies, can elicit different responses [7,29,30]. For instance, low-frequency mobilization NDTs may increase systolic blood pressure, whereas high-frequency NDTs can have the opposite effect [21]. According to Alharmoodi et al. [27], tensioner NDTs can potentially exert more strain on the peripheral nervous system and ANS compared to slider NDTs. However, additional research is required to draw comprehensive conclusions, as no randomized controlled clinical trials have directly compared the effects of slider and tensioner NDTs on SNS activity. Moreover, the authors are not aware of any published studies investigating the mechanical differences between slider and tensioner neural mobilization techniques and their effect on the SNS activity. Exploring this aspect could significantly enhance our understanding of the descending pain pathways, particularly those involving the periaqueductal gray matter. Such insights may elucidate the mechanisms underlying the analgesic effects of NDTs [12,31]. The sympathetic slump position (SSP) is characterized by a seated posture with forward head flexion, thoracic flexion, and lumbar extension. This posture is known to cause mechanical and physiological stress on the nervous system, leading to increased SNS activity [21,32]. Research indicates that assuming this particular posture triggers physiological responses that are characteristic of sympathetic arousal, such as increased heart rate and blood pressure. Therefore, this posture is an appropriate setting for evaluating the effect of NDTs on SNS activity.

Applying NDTs to the SSP allows researchers to evaluate the effect of these techniques on SNS function under conditions of increased sympathetic activity. This posture mimics real-world scenarios that elevate sympathetic activation, like stress or pain. Researchers can use NDTs in this context to monitor changes in SNS indicators, including heart rate variability, skin conductance (SC), and blood flow. This allows them to gain insights into the effectiveness of these techniques in reducing sympathetic arousal and improving overall nervous system function. This approach facilitates comprehension of the therapeutic benefits of NDTs in the management of conditions associated with heightened sympathetic activity, such as chronic pain and anxiety [27].

Despite the recognized importance of neurodynamic techniques in clinical practice, direct comparisons of their effects on the SNS, particularly in asymptomatic individuals, are limited. This study addresses this gap by comparing the effects of two NDTs—slider versus tensioner NDTs—on SNS activity. This study aims to establish a baseline of SNS responses in a controlled setting by comparing the effects of two neurodynamic mobilization techniques—slider versus tensioner NDTs—on SNS activity in a posture known to elevate sympathetic responses. These findings will require further validation in symptomatic populations to determine clinical relevance and guide more effective interventions for conditions linked to altered autonomic regulation. It is hypothesized that while both techniques modulate SNS activity, tensioner NDTs will have a more pronounced effect on the SNS due to the increased mechanical load, whereas slider NDTs may produce a more gradual modulation due to their dynamic nature.

## 2. Materials and Methods

### 2.1. Study Design

We conducted a study using a randomized, double-blind, sham-controlled, parallel-group design. The recruitment process spanned from March 2023 to May 2023, and the randomized controlled trial lasted for five months, from June to October 2023, at the research laboratory of European University Cyprus. The study received ethical approval from the Cyprus National Bioethics Committee (EEBK/EΠ/2021/58), adhered to the guidelines outlined in the Declaration of Helsinki, and was registered on ClinicalTrials.gov (identifier: NCT06098131). A similar data collection protocol was employed by other authors [28].

### 2.2. Sample Size

The sample size for this study was calculated based on previous studies [33,34] that reported changes in SC values among experimental groups. We anticipated detecting a minimum of an 8% difference in SC values from baseline, which was based on observed effect sizes in these prior studies. The calculation assumed a pooled standard deviation of 10% [35], a power of 80%, and an alpha level of 0.078 to ensure sufficient statistical sensitivity. Initially, we determined that 75 participants would be necessary to achieve this power; however, to adjust for an expected 20% dropout rate, we increased the number per group to 30, resulting in a total of 90 participants across the three groups. This adjustment ensures that the study maintains adequate power to detect clinically meaningful differences, even with potential attrition.

### 2.3. Participants

This study enrolled ninety healthy, pain-free individuals aged 18–40, from both genders, with a body mass index (BMI) ≤ 30, to minimize confounding effects related to altered SNS regulation [30] and specific age-related pathologies. The age range was specifically chosen to exclude minors, as recruiting participants younger than 18 can complicate consent issues, as outlined by Kuther [23], and to avoid the inclusion of older adults who might have undiagnosed age-related conditions such as asymptomatic disc pathologies, which are more prevalent in individuals over 40 [36,37]. This strategic choice ensures a homogeneous sample, free from the complexities associated with developmental and degenerative changes that could skew the fundamental understanding of NDT effects on SNS activity. By using healthy participants within this age range, we established a baseline response to NDTs, enhancing our understanding of their physiological impacts on sweat response, temperature, and blood flow. This baseline is crucial for distinguishing the inherent effects of NDTs from those influenced by specific health conditions and provides foundational data for future comparisons in individuals with neurological or musculoskeletal disorders.

Participants were carefully selected based on their health status: those with a history of low back pain, skin disorders, previous manual therapy, lower limb injuries, or chronic systemic health conditions such as diabetes were excluded. Additional exclusion criteria included recent consumption of food, caffeine, nicotine, or alcohol, engagement in strenuous activity within three hours prior to the study, and use of medications that could affect the SNS. All participants were informed about the study’s objectives and methodologies, completed a medical questionnaire, and provided written consent. Anthropometric measurements and eligibility details are summarized in Table 1, ensuring a homogeneous sample for accurate assessment of NDTs’ effects on the SNS. 

### 2.4. Interventions

**Figure 1:**
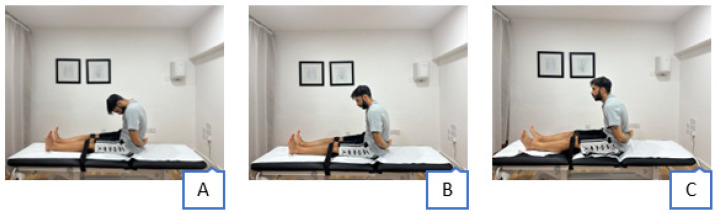
Neural mobilization techniques. (**A**) Tensioner Technique. (**B**) Slider Technique. (**C**) Control group.

Participants were assigned to one of the three experimental conditions during the intervention (Figure 1). The experimental interventions employed were analogous to those used by other authors [28]. The objective of the slider technique group was to increase nerve excursion through coordinated movements. Participants attained the SSP by maintaining an upright posture with their spine straight and knees fully extended. They also clasped their hands behind their bodies and flexed their head and thoracic spine to the maximum extent. The SSP is specifically designed to apply tension to the neural structures, potentially affecting SNS activity by influencing nerve tension and mobilization. This position is comparable to the conventional slump test, but it focuses on the particular posture and movement patterns that are important for achieving the objectives of the slider technique in neurodynamic interventions. Additionally, the sacral block and fixation belt were used to standardize participant positioning and minimize any potential movement during the interventions that could otherwise influence the outcomes. Sacral stability was achieved by using a sacral block and fixation belt to maintain the same knee extension [1]. These tools are essential for maintaining consistency across participants, thereby ensuring the reliability of the data collected. Participants received NDTs according to a standardized protocol, which involved performing three sets of one-minute slider movements with one-minute rest intervals between each set. Participants in the sliding technique group first adopted the SSP. The therapist maintained the head in a less-flexed and closer-to-neutral position. During this process, they performed full dorsiflexion of the foot, ensuring that the movements allowed the nerve to glide through its pathway rather than creating significant tension [1,38].

Participants in the tensioner technique group first adopted the SSP, then proceeded to enhance neural tension by simultaneously fully dorsiflexing the foot and fully flexing the cervical spine forward, thereby increasing tension throughout the nerve pathway [1]. The technique is applied dynamically with relaxation intervals to prevent any potential negative effects on neural vasculature. This process was repeated three times, with a one-minute rest interval between each iteration, to ensure uniformity in both groups (Figure 1). A similar data collection protocol was employed by other authors [28]. The participants in the control group maintained a seated position that closely resembled the SSP for a duration of 20 min without receiving any specific maneuvers or interventions. This position involves sitting with a straight spine and hyperextended knees while clasping the hands behind the body.

The modified SSP in the control group serves as a reference point for evaluating the dynamic effects of NDTs on sympathetic activity. Maintaining a static posture without maneuvers isolates the specific effect of the neurodynamic movements. By incorporating sacral stability, the potential influence of posture-related factors is accounted for, thereby improving the accuracy and reliability of the results. This setup allows researchers to discern the distinct effects of NDTs on sympathetic activity, enhancing our comprehension of their therapeutic effects [Supplementary Materials].

For our study, we conducted three series of one-minute neurodynamic movements to ensure reliability and consistency in our measurements. This methodology allows for a robust assessment of the effect of slider and tensioner techniques on sympathetic activity [39]. Repeating the measurements allows us to detect any potential variability or fluctuations in response throughout the duration of the intervention [28]. By calculating the mean of the outcomes from these repetitions, we obtain a more accurate depiction of the overall response to the NDTs [29].

### 2.5. Randomization and Blinding

Two days prior to the experiment, a randomization process assigned 90 participants to three intervention groups using a Stat Trek online number generator (https://stattrek.com/statistics/random-number-generator). The randomization process was designed to ensure equal distribution of participants based on age, gender, and BMI to maintain homogeneity across the groups. This stratified randomization helped control for potential confounding variables that could influence the study outcomes [39,40]. The outcomes of this process were securely stored in a sealed envelope, which was only accessed by the therapist at the onset of data collection.

**Figure 2:**
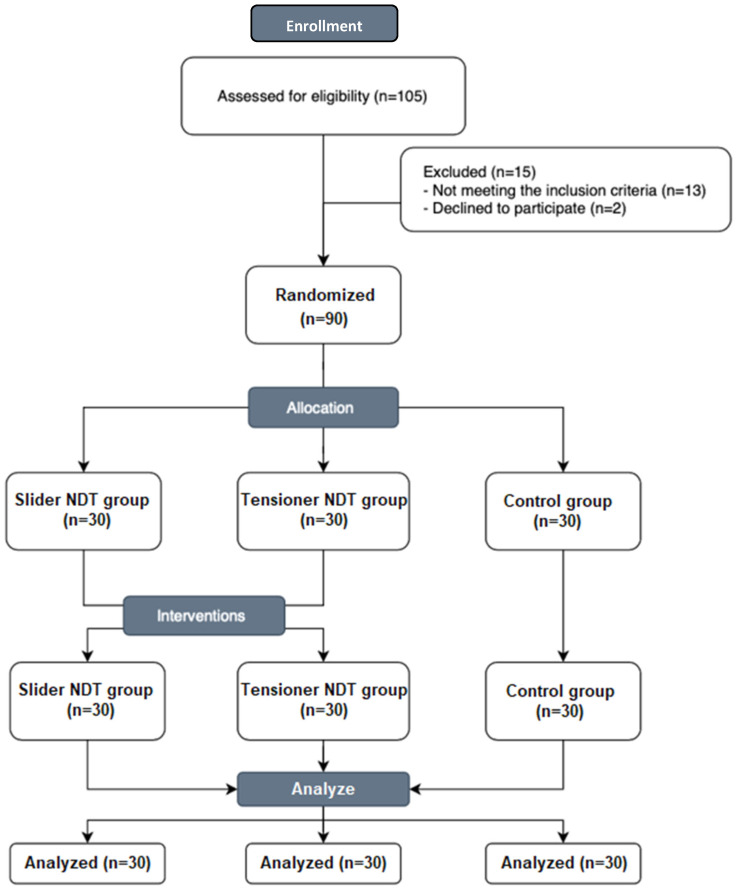
CONSORT flow diagram.

To preserve the integrity of the double-blind study design, both the evaluator and the participants were kept unaware of the group assignments. The evaluator, who recorded all outcome measures, did not have access to the randomization details, which were known only to the therapist until the study commenced (Figure 2). This approach minimized any potential bias, with participants also uninformed about their specific group assignments to further reduce expectation bias [41].

### 2.6. Laboratory Assessments

Participants visited the laboratory once, where they were given instructions to abstain from any interventions and maintain regular activity before the visit. They also had a familiarization session before the data collection. The visit consisted of a stabilization phase, baseline recording, randomized control (sham treatment), slider, or tensioner intervention.

### 2.7. Instrumentation and Measurements

SC responses were recorded by placing two disposable electrodes (EL507, Biopac Systems, Inc., Goleta, CA, USA) on the plantar surface of the second and third toes on both feet. These electrodes captured an electrical signal that was proportional to the changes in SC. The skin was prepared in accordance with the standard protocol for Biopac measurement [33,34]. The data acquisition system used in this study was the Biopac MP36 Data Acquisition Unit (Biopac Systems Inc., Goleta, CA, USA). It was equipped with a galvanic skin response 100B electrodermal activity amplifier, capable of analyzing the electrodermal activity within a range of 0 to 35 and recording SC (measured in μMhos) at a rate of 200 samples per second. The collected response data were subsequently transferred to a personal computer for storage and analysis using the Biopac Student Lab Pro Works version 4.1 software. To normalize between-participant differences in SC activity levels, we used the percentage change method, as described by Perry and Green [42] and utilized in other studies [43]. This approach calculates changes relative to individual baselines, which is essential for accommodating variations in initial SC levels among participants. Normalizing data in this manner ensures that observed changes are attributable to the intervention and not to baseline disparities. The skin temperature was measured before and after the intervention using a thermo-camera (ThermaCam SC2000©, FLIR, Danderyd, Sweden). The thermo-camera was oriented to capture images of each participant’s entire body during the initial measurement phase. An ambulatory blood pressure monitor (Omron© HEM-9210T Healthcare, Co. Ltd., Kyoto, Japan) was used to continuously track the participant’s blood pressure and heart rate. The blood pressure cuff was positioned on the participant’s left arm throughout the intervention and until the conclusion of the rest period. Our study utilized well-established measurement techniques that have undergone prior validation for their reliability. In order to maintain precision in our analysis, we converted changes in blood pressure and body temperature into percentages using the formula employed by previous researchers [44]. This formula is widely utilized in scientific research because of its effectiveness in representing changes in blood pressure and other physiological measurements.

### 2.8. Procedures

The study was conducted in a temperature-controlled room (ranging from 23 °C to 26 °C) [21]. Prior to data collection, participants’ age, height, gender, and BMI were recorded, following the provision of informed consent. Data collection and physiotherapist treatment remained consistent throughout the entire five-month duration of the study. To maintain the integrity of the double-blinding, a screen was utilized to create a physical barrier between the treatment area (plinth) and the data collector. This approach was implemented to ensure that the data collector remained unaware of the treatment-allocation group for each participant. The data collector was the only person who had access to the Biopac software, blood pressure monitor, and thermo-camera to ensure that the therapist remained blinded to the study results. The therapist remained hidden behind the screen for the entire duration of the experiment. The therapist and data collector had three years of clinical experience in physiotherapy, with a specialization in manual therapy.

**Figure 3:**
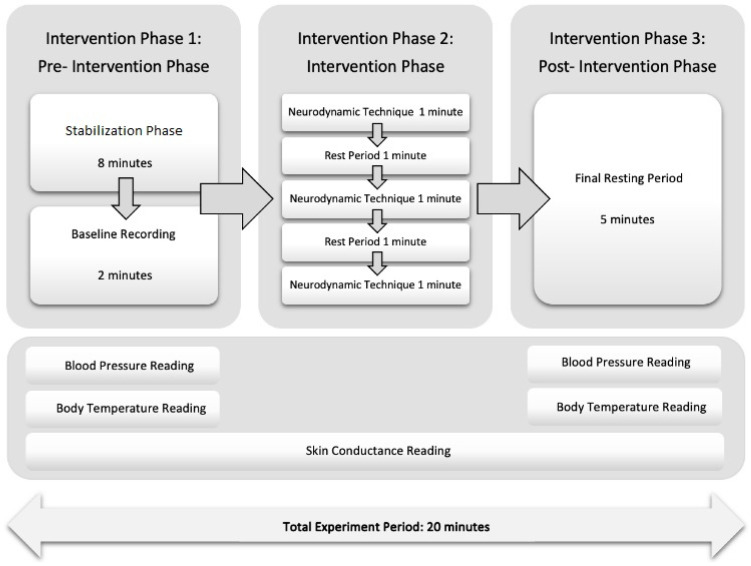
Schematic representation of the study protocol.

The experiment consisted of three phases (Figure 3). During the pre-intervention phase, participants started with an eight-minute stabilization period to achieve a physiological resting state. This was followed by a two-minute baseline recording period, during which the therapist recorded body temperature and blood pressure. The intervention phase consisted of three one-minute intervention periods, with each period being followed by a one-minute rest period. The designated intervention leg, specifically the left leg, was uniformly used across all participants to ensure consistency and reduce variability. This standardization helped systematically assess any bilateral or global effects, facilitating a clearer analysis of the systemic effect of the neurodynamic intervention. The experiment concluded with a five-minute resting period in the post-intervention phase, aligning with established protocols designed to allow physiological parameters to return to baseline [28].

SC was continuously recorded throughout the 20-minute experiment. The data collector provided verbal cues, specifically “Intervention Phase 1”, “Intervention Phase 2”, and “Intervention Phase 3”, and markers were placed on the Biopac graph to indicate phase transitions. A similar data collection protocol was employed by other authors [28]. During the post-intervention phase, body temperature and blood pressure were recorded. When the participant assumed the resting position, the data collector promptly verified SC activity using the thermo-camera, ambulatory blood pressure monitor, and Biopac graph readings.

### 2.9. Statistical Analysis

Data are presented as mean ± standard deviation (SD). Prior to analysis, assumptions of normality and homogeneity of variance were verified using Shapiro–Wilk and Levene’s tests, respectively. These tests confirmed that all variables except blood pressure followed a normal distribution, supporting the use of parametric tests for the analysis. The choice of a three-way mixed-measures ANOVA (3 intervention levels × 3 experimental period levels × 2 sides) was driven by the study design, which included repeated measures over time (pre-intervention phase, intervention phase and post-intervention phase), interventions (tensioner NDT, slider NDT and control), and a comparison across two physiological sides (left and right). A two-way mixed-design ANOVA was performed to examine the main effects and interactions of treatment (tensioner NDT, slider NDT and control) and sides (left and right) on mean subject-based dependent measures. This was chosen to specifically analyze the treatment effects over time without the interaction of side, providing a focused view of the temporal dynamics of the interventions.

The non-parametric Kruskal–Wallis one-way analysis was used for non-normally distributed data. The significance level was set at *p*  <  0.05. In this study, effect sizes calculated using Cohen’s d are classified as small (d < 0.5), medium (d = 0.5–0.8), and large (d > 0.8), providing a framework for assessing the clinical significance of the findings [36]. All statistical analyses were conducted using SPSS (version 24; IBM, Chicago, IL, USA). Effect size calculations were performed using Microsoft Excel 365 (Microsoft Corporation, Redmond, WA, USA, https://www.microsoft.com/en-us/microsoft-365/excel).

## 3. Results

Table 1 displays the baseline demographic characteristics, including gender, age, height, weight, and BMI, as well as the assessment results (measured at room temperature) for the three groups. The statistical analysis, including Levene’s test, indicated no significant differences in these variables among the groups (*p* > 0.05). Specifically, the one-way repeated-measures ANOVA demonstrated non-significant variations in room temperature (*p* = 0.51) within thresholds considered inconsequential for percentage changes in SC [36].

### 3.1. Baseline-to-Intervention Phase

The statistical analysis showed that for the percentage changes in SC, the two-way interaction of intervention phase × side did not have a significant effect. However, as expected, there was a significant two-way interaction for the intervention phase × treatment group [F(2,174) = 41.3; *p* < 0.001]. Similarly, the two-way interaction of intervention phase × side did not have a significant effect. However, as expected, there was a significant two-way interaction for the intervention phase × treatment group [F(2,174) = 41.3; *p* < 0.001].

The main effects indicated statistically significant differences (*p* < 0.05) in the intervention phase [F(2,174) = 338.53; *p* = < 0.001], between sides [F(1,174) = 5.59; *p* < 0.019], and between treatment groups [F(2,174) = 13.10; *p* < 0.001] (see Table 2). For the follow-up test, we examined mean differences in percentage changes in SC between the intervention phase and treatment group for each leg.

As shown in Table 2, post hoc Bonferroni analysis revealed significantly higher percentage changes in SC for both the slider and tensioner groups, compared to the control group, during the left-leg baseline-to-intervention period (slider vs. control [F(2,174) = 44.06; *p* < 0.001 and d = 1.14] and tensioner vs. control [F(2,174) = 44.06; *p* < 0.001 and d = 2.17]). No significant difference was observed between the slider and tensioner groups in the left-leg percentage changes in terms of the percentage change in SC [F(2,174) = 44.06; *p* = 0.13 and d = 0.44]. Similarly, the tensioner group demonstrated a significant effect compared to the control group [F(2,174) = 1.85; *p* < 0.001 and d = 1.76] on the right leg. Furthermore, the slider group had a significant effect on the right leg compared to the control group [F(2,174) = 1.85; *p* < 0.001 and d = 1.66]. However, no significant difference was observed between the slider and tensioner groups for the right leg in terms of percentage changes in SC [F(2,174) = 1.85; *p* = 1.00 and d = 0.07].

### 3.2. Intervention Phase to the Final Resting Phase

As shown in Table 3, the post-hoc Bonferroni analysis conducted between the intervention and final resting phase revealed significant inhibitory differences in percentage changes in SC among groups. Both the slider and tensioner groups demonstrated significantly higher inhibitory effects on the left leg compared to the control group (slider vs. control: [F(2,89) = 7.82; *p* < 0.001, d = 1.20]; tensioner vs. control: [F(2,89) = 18.02; *p* < 0.001, d = 1.64]). No statistically significant difference was observed between the slider and tensioner groups in terms of the percentage changes in SC for the left leg (d = 0.22). The tensioner group exhibited a significant inhibitory effect on the right leg compared to the control group [F(2,174) = 20.21; *p* < 0.001 and d = 0.73]. Similarly, the slider group showed a significant inhibitory effect compared to the control group [F(2,174) = 18.21; *p* < 0.001 and d = 1.47]. The study did not find any statistically significant difference between the slider and tensioner groups in terms of the percentage changes in SC for the right leg [F(2,174) = 20.21; *p* = 0.12 and d = 0.47].

For the percentage change in blood pressure, the Kruskal–Wallis H Test revealed no statistically significant differences between the three groups for the systolic blood pressure [H(2) = 0.11; *p* = 0.95] and the diastolic blood pressure [H(2) = 5.54; *p* = 0.06] (Table 3).

For the percentage change in body temperature, the one-way independent-measures ANOVA revealed a significant overall F-ratio [F(2,87) = 15.22; *p* < 0.001]. Post-hoc Bonferroni analysis revealed significant differences between the control group and slider group [F(2,87) = 15.22; *p* < 0.001 and d = 0.95], as well as the control and tensioner group [F(2,87) = 15.22; *p* < 0.001 and d = 1.48] (Table 3).

### 3.3. Baseline Phase to the Final Resting Phase

For percentage changes in SC, as shown in Table 4, the 3 × 2 mixed-measures ANOVA revealed a significant group × site interaction [F(3,174) = 16.4; *p* < 0.001]. Both the slider and tensioner groups exhibited significantly higher inhibitory effects on the left leg when compared to the control group (slider vs. control [F(2,89) = 18.02; *p* < 0.001 and d = 0.38], and tensioner vs. control [F(2,89) = 18.02; *p* < 0.001 and d = 1.08]). There was no statistically significant difference in percentage changes in SC between the slider and tensioner groups for the left leg [F(2,89) = 18.02; *p* = 1.00 and d = 0.89]. The slider group showed a significant inhibitory effect on percentage changes in SC compared to the control group on the right leg [F(2,89) = 18.02; *p* < 0.001 and d = 0.48], as well as tensioner vs. control [F(2,89) = 18.02, *p* < 0.02 and d = 0.77]. No significant difference was found in terms of percentage changes in SC between the slider and tensioner groups for the right leg [F(2,89) = 18.02; *p* = 0.12 and d = 0.36].

## 4. Discussion

This study examined the effect of NDTs on the SNS activity level. In order to achieve this objective, we compared the effects induced by three different conditions—slider, tensioner, and control—on asymptomatic participants who were positioned in the SSP. This comparison was carried out during two specific time periods: from the baseline to the intervention phase and from the intervention phase to the end rest period. The main findings were that (a) both slider and tensioner NDTs showed no significant difference in the percentage changes in SC, blood pressure, and body temperature, and (b) no side-specific response was noted. This study is the first to examine the effects of slider and tensioner NDTs on SNS activity levels, blood pressure, and temperature.

However, it is important to consider these findings in the context of the existing literature. Although no previous study has directly compared percentage change in SC, body temperature, and blood pressure between these specific NDT approaches, similar studies have explored the effects of different NDTs on neural function. Previous studies [2,8] demonstrated significant biomechanical differences between slider and tensioner NDTs. These studies found that the slider NDT produced greater nerve stimulation and excursion compared to the tensioner NDT. However, these studies were conducted on cadaveric models, which lack the physiological responses necessary to exhibit changes in the SNS, such as SC and skin temperature. This methodological difference might explain why our study, using live human participants, found no significant difference between the two NDTs concerning SNS activity levels.

The SC response, indicative of SNS activity and arousal, is quantified through changes in the electrical conductivity of the skin, a result of increased sweat gland activity [37]. Our study monitored SC changes over two distinct periods: from baseline to the intervention phase and from the intervention to the end rest period. Both the slider and tensioner experimental groups demonstrated significantly higher SC changes compared to the control group during these periods, indicating enhanced SNS activation. Specifically, the increase from baseline to intervention for the left and right legs was statistically significant (left leg: *p* < 0.001, right leg: *p* < 0.001), as was the increase from intervention to end rest (left leg: *p* < 0.001, right leg: *p* < 0.02). Despite these significant changes, the difference between the two NDTs was not statistically significant from baseline to final rest (*p* = 0.12) and from intervention to final rest (*p* = 0.12). However, the tensioner group demonstrated a larger effect size than the slider group compared to the control group. This suggests that while both techniques are effective, the tensioner technique may induce a more pronounced sympathoexcitatory response [21,45]. These findings underscore the potential for slider and tensioner NDTs to activate the SNS robustly, with the tensioner technique, in particular, showing a capacity to rapidly trigger fight-or-flight processes linked to increased dPAG activation [18,46].

In addition, our results are consistent with those of Alshami et al. [31], who also found no significant differences in SNS activation between slider and tensioner NDTs. However, it is worth noting that some earlier studies, particularly those using cadaveric models, reported differences between the techniques. These discrepancies could stem from the fact that cadaveric studies lack the physiological responses, such as changes in SC and skin temperature, which are critical in evaluating SNS activity. The lack of side-specific responses in our study further supports the idea that NDTs elicit a global effect on the SNS, which contrasts with some studies that have reported more localized effects, particularly those focusing on biomechanical aspects rather than autonomic responses.

The observed increase in percentage changes in SC in the slider and tensioner groups compared to the control group could be due to an ANS response during both the baseline-to-intervention phase and the intervention to end rest period [15,40]. This indicates that both NDTs induce SNS activation, as reflected in the heightened SC levels [21,46]. However, the tensioner technique had a greater effect. This could be attributed again to the rapid activation of fight-or-flight processes associated with increased dPAG activation [18,39]. Gauriau and Bernard [41] suggested that increased dPAG activation might cause sympathoexcitatory responses in noradrenergic systems, which could lower substance-P release and mitigate mechano-nociceptive stimuli. This effect may contribute to a more robust analgesic effect, which may be beneficial in chronic pain management [42,43]. However, the absence of a statistically significant difference between the two NDTs suggests that neither technique is superior.

Furthermore, Slater et al. [39] found significant changes in percentage change in SC following neuromobilization techniques, which supports our findings of increased SC in both the slider and tensioner groups. However, the exact mechanisms behind these changes remain unclear, especially given the lack of significant differences between the techniques. This suggests that while NDTs can influence SNS activity, the specific technique may not be as crucial as previously thought. These mixed results across studies highlight the need for further research, ideally combining biomechanical and physiological analyses, to better understand the relationship between different NDT types and their effects on the SNS.

Our study found an increase in body temperature from baseline to the final resting phase. This indicates a potential autonomic response associated with heightened sympathetic activity [40]. Nevertheless, previous research suggests that skin temperature variations are location-dependent, a challenge we addressed by strategically positioning the thermo-camera to capture the participant’s entire body [44]. The absence of a statistically significant difference in blood pressure between the slider and tensioner groups suggests that both NDTs do not affect cardiovascular parameters [30,45].

These findings are consistent with prior studies [27,40,46,47,48,49] that have also reported SNS activation following the use of NDTs. Alshami et al. [31] also found no significant differences in SNS activation between slider and tensioner NDTs. However, these results contradict other studies indicating differences between the techniques [1,8]. This discrepancy could be attributed to the use of cadaveric models in most studies, which lack the physiological responses necessary to exhibit changes in the SNS, such as changes in SC and skin temperature [2,50,51]. As a result, these studies may have limitations that affect the applicability of their results to clinical practice.

A second significant finding from our study was the absence of side-specific responses, which points to a global effect of NDTs. This phenomenon, well-recognized in the field of physiotherapy, suggests that NDTs not only focus on localized treatment areas but also elicit systemic benefits that permeate multiple physiological functions, such as posture improvement, pain modulation, and enhanced neuromuscular coordination [51]. This observation aligns with existing studies [21,52,53,54], as our results showed no statistically significant differences in SC changes between the left and right legs across all intervention phases (*p* = 0.12), underscoring the comprehensive effect of NDTs. The broader implications of these findings suggest that NDTs induce extensive neurophysiological responses that transcend simple mechanical interactions, affecting nerve conductivity and neurotransmitter dynamics across the nervous system [39]. Such effects illustrate a complex interplay between NDTs and neurophysiological processes, potentially impacting overall health and recovery. These systemic responses to NDTs, as reported in previous studies [26,55], highlight their role in influencing SNS activity and broader physiological parameters, reinforcing the notion of NDTs as a multi-faceted intervention tool. Understanding these global effects is crucial, not only for appreciating the therapeutic breadth of NDTs, but also for harnessing their full potential in clinical practice. This knowledge equips physiotherapists with the ability to apply these techniques more strategically across various clinical scenarios [56,57]. By modulating systemic physiological responses, NDTs offer a versatile approach to managing conditions characterized by altered autonomic regulation, such as chronic pain and stress-related disorders. Future research should continue to explore these interactions, aiming to elucidate the long-term clinical outcomes of regular NDT application and to refine treatment protocols that maximize patient benefits across diverse health spectrums.

All outcome measures that were collected successfully proved that NDTs can activate the SNS. This activation is justified, based on the existing literature, since neurodynamics can affect the activation of the SNS in several ways. Research has shown that the release of neurotransmitters, such as norepinephrine and dopamine, can trigger the SNS response [53]. Neurotransmitters are the chemical messengers of the nervous system and are essential in transmitting signals between neurons [4]. When neurotransmitters are released into the synaptic cleft, they activate specific receptors on neighboring neurons and trigger a cascade of events that can ultimately lead to the activation of the SNS [4,54]. Moreover, according to Schmelz [58] and Thacker et al. [59], activation of the dPAG can quickly activate several fight-or-flight processes. This can activate the sympathoexcitation response in the noradrenergic systems, which can inhibit the Substance-P release and subsequently suppress the mechano-nociceptive stimuli [60]. This can additionally justify the activation on the SC (slider NDT 42.31% changes on the left leg and 40.45% on the right leg; and tensioner NDT 57.33% changes on the left leg and 38.67% on the right leg), which was noted in the present study.

Furthermore, the architecture of the nervous system can also affect how the SNS is activated [61]. A series of ganglia along the spinal cord make up the sympathetic nervous system. The hypothalamus and other brain regions can send signals to these ganglia, which are linked by nerve fibers [62]. The SNS is activated by the chromaffin cells, which are specialized cells in the ganglia that release adrenaline and noradrenaline straight into the circulation [63]. This can provide an explanation for the increase in blood pressure that was observed in the current study. More specifically, following the NDTs, a higher value was observed in the systolic blood pressure and tensioner NDT group compared to the control group.

Regardless of whether individuals are asymptomatic and healthy or experiencing pathological conditions, it is expected that both groups would react similarly when influenced by external forces such as NDTs [21]. It is argued that the response after the introduction of NDTs remains comparable among asymptomatic populations, suggesting that differences or variations in outcomes may arise from the tasks assigned to research participants and the specific context of NDT implementation [64,65,66,67]. Moreover, Slater et al. [39], who conducted similar research on asymptomatic healthy individuals, emphasize the importance of initially assessing the effects of NDTs on the SNS activity within an asymptomatic population before applying them to a clinical population. This approach is considered crucial to understanding the potential implications and safety of applying these techniques before exploring their effect on a clinical population. This foundational knowledge not only adheres to ethical research practices but also sets benchmarks for physiotherapeutic interventions [68,69,70,71,72].

The effect of touch on the SNS involves complex physiological responses that can influence autonomic balance and emotional states. Research has shown that gentle touch, such as massage or therapeutic touch, can decrease sympathetic activity, resulting in physiological changes such as lowered heart rate, reduced blood pressure, and decreased levels of stress hormones like cortisol [61,62,68,73,74,75]. Incorporating neural-dynamic NDTs alongside touch-based interventions may offer additional avenues for modulating sympathetic activity, thereby expanding therapeutic strategies aimed at enhancing autonomic balance and emotional well-being.

### 4.1. Limitations

While considerable efforts were made to control external factors that could lead to false activation of the SNS, including a diverse gender sample to minimize bias from estrogen levels and menstrual cycles [63], excluding women would have limited the generalizability of the results. Additionally, the sample had a relatively low average age, restricting the applicability of findings to the age range of 18-to-40 years only.

Another limitation is the absence of follow-up assessments, which restricts our understanding of the long-term effects of NDTs on SNS activity [64,65,66,76]. The short duration of the study means that while immediate effects were documented, the persistence of these effects over time remains unknown. This limitation affects the ability to ascertain the durability of the sympathetic responses and the potential cumulative benefits or drawbacks of repeated NDT sessions. However, this was not the aim of our study. Future research should extend these investigations to include longitudinal studies that can provide insights into the duration and stability of the effects observed, as well as explore the relationship between sustained changes in SNS activity and the analgesic effects of NDTs.

### 4.2. Practical Application

This study demonstrates that both the slider and tensioner techniques have similar effects on asymptomatic individuals. However, it is important to note that these results cannot be directly applied to symptomatic populations. Nevertheless, research on asymptomatic individuals is crucial for obtaining essential baseline data. Understanding neural mobilization effects in asymptomatic populations helps physiotherapists develop theoretical frameworks and identify potential benefits. These insights suggest that neural mobilization might have widespread advantages, encouraging further research into its effects on symptomatic patients. The findings contribute to evidence-based practice, aiding clinical reasoning and decision-making. While direct application to symptomatic patients requires caution, this research offers a foundation for developing and testing hypotheses. Future studies are required to validate these findings in clinical populations; however, the importance of studying asymptomatic individuals in advancing clinical practice is evident.

## 5. Conclusions

The present study demonstrated that both slider and tensioner NDTs significantly influence SNS response, as shown by measures of SNS activation, blood pressure, and body temperature in asymptomatic participants. Meanwhile, the tensioner group showed a more substantial effect, indicating superiority.

These findings suggest that both techniques have potential utility in physiotherapy for modulating systemic physiological responses. However, as these results are based on asymptomatic individuals, further validation is needed before applying them to clinical populations. Clinicians should use these techniques cautiously and await further research. Comprehensive clinical trials are required to confirm their effectiveness and safety in symptomatic populations, assess long-term benefits, and establish their clinical value. Future research should focus on longitudinal studies and include diverse patient demographics to fully understand the clinical implications of NDTs.

## Figures and Tables

**Table 1:** Baseline values of sex, age, weight, height, BMI, and room-temperature measurements of the three groups. Values are expressed as mean ± SD.

Variable	Slider Technique Group (n ^a^ = 30)	Tensioner Technique Group (n = 30)	Control Group (n = 30)	*p*-Value
Sex (Male/Female)	53%/47%	53%/47%	47%/53%	-
Age (Years)	21.70 ± 2.63	26.30 ± 3.17	27.00 ± 3.16	0.11
Weight (kg ^b^)	73.20 ± 7.92	73.10 ± 7.97	73.80 ± 7.95	0.29
Height (cm ^c^)	173.80 ± 1.79	173.10 ± 1.78	174.40 ± 1.79	0.59
BMI ^d^ (kg/cm^2 e^)	23.70 ± 2.51	23.80 ± 2.54	24.00 ± 2.53	0.35
Room temperature (°C ^f^)	24.70 ± 2.51	24.60 ± 2.49	24.60 ± 2.51	0.51

^a^ n: number of partitions; ^b^ kg: kilogram; ^c^ cm: centimeter; ^d^ BMI: body-mass-index; ^e^ kg/cm: kilogram force per square centimeter; ^f^ °C: degrees Celsius.

**Table 2:** Skin conductance values for the slider, tensioner, and control groups for the left and right legs from the baseline phase to the intervention phase. Values are presented as mean ± SD.

Side	Outcome Measures	Slider Group	Tensioner Group	Control Group	*p*-Value
Left Leg	Percentage change in skin conductance (μMhos ^a^)	42.31 ± 38.27	57.33 ± 28.64	10.03 ± 11.17 ^bc^	<0.001
Right Leg	Percentage change in skin conductance (μMhos ^a^)	40.45 ± 26.78	38.67 ± 23.16	4.93 ± 14.08 ^bc^	<0.001

^a^ μMhos: mean anticipatory and task-activity skin conductance level; ^b^ denotes significant difference compared to slider group; ^c^ denotes significant difference compared to control group.

**Table 3:** Outcome-measure values from the intervention phase to the final resting phase for the slider, tensioner, and control groups for the left and right legs. Values are presented as mean ± SD.

Side	Outcome Measures	Slider Group	Tensioner Group	Control Group	*p*-Value
Left Leg	Percentage change in skin conductance (μMhos ^a^)	−34.21 ± 23.77	−39.18 ± 20.08	−10.57 ± 14.14 ^bc^	<0.001
Right Leg	Percentage change in skin conductance (μMhos ^a^)	−38.62 ± 22.53	−26.58 ± 27.56	−10.02 ± 15.65 ^bc^	<0.001
Percentage change in Systolic Blood Pressure (mmHg ^d^)		4.55 ± 0.11	4.55 ± 0.11	4.55 ± 0.11	0.95
Percentage change in Diastolic Blood Pressure (mmHg ^d^)		4.65 ± 0.14	4.65 ± 0.14	4.65 ± 0.14	0.06
Percentage change in Body Temperature (°C ^e^)		36.92 ± 0.39	36.99 ± 0.36	36.81 ± 0.43 ^bc^	<0.001

^a^ μMhos: mean anticipatory and task-activity skin conductance level; ^b^ denotes significant difference compared to slider group; ^c^ denotes significant difference compared to control group; ^d^ mmHg: millimeters of mercury; ^e^ °C: degrees Celsius.

**Table 4:** Outcome-measure values from the baseline phase to the final resting phase for the slider, tensioner, and control groups for the left and right legs. Values are presented as mean ± SD.

Side	Outcome Measures	Slider Group	Tensioner Group	Control Group	*p*-Value
Left Leg	Percentage change in skin conductance (μMhos ^a^)	30.77 ± 10.49	42.89 ± 11.83	11.37 ± 2.35 ^bc^	<0.001
Right Leg	Percentage change in skin conductance (μMhos ^a^)	36.09 ± 7.94	−30.44 ± 14.87	9.6 ± 0.4 ^bc^	<0.001

^a^ μMhos: mean anticipatory and task-activity skin conductance level; ^b^ denotes significant difference compared to the slider group; ^c^ denotes significant difference compared to control group.

## Data Availability

All files are available from the Figshare database (https://doi.org/10.6084/m9.figshare.25185011.v1), and the data are open-access.

## Supplementary Materials

The following supporting information can be downloaded at: https://www.mdpi.com/article/10.3390/jcm13175098/s1.
