# Correction: Papacharalambous et al. Comparative Effects of Neurodynamic Slider and Tensioner Mobilization Techniques on Sympathetic Nervous System Function: A Randomized Controlled Trial. *J. Clin. Med.* 2024, *13*, 5098

**DOI:** 10.3390/jcm14124202

**Published:** 2025-06-13

**Authors:** Charalambos Papacharalambous, Christos Savva, Christos Karagiannis, Eleftherios Paraskevopoulos, George M. Pamboris

**Affiliations:** 1 Department of Health Sciences, School of Sciences, European University Cyprus, Nicosia 2404, Cyprus; c.karayiannis@euc.ac.cy (C.K.); g.pamboris@euc.ac.cy (G.M.P.); 2 Department of Life and Health Sciences, Frederick University, Limassol 3080, Cyprus; c.savva@frederick.ac.cy; 3 Department of Physiotherapy, University of West Attica, 122 43 Athens, Greece; elparaskevop@uniwa.gr

## Errors in Tables

In the original publication [1], there was a mistake in Tables 1 and 3. In Table 1, the standard deviation baseline values for demographic and environmental variables were not presented accurately. In Table 3, the mean and standard deviation for percentage change in systolic and diastolic blood pressure were not presented correctly. The corrected Table 1 and Table 3 appear below.

## Text Correction

**Changes to Section 2.9. Statistical Analysis**

The fourth sentence should be corrected to “A two-way mixed-design ANOVA was performed to examine the main effects and interactions of treatment (tensioner NDT, slider NDT and control) and sides (left and right) on mean subject-based dependent measures.”

**Changes to Section 3.1. Baseline-to-Intervention Phase**

The first paragraph should be corrected to read: “The statistical analysis showed that for the percentage changes in SC, the two-way interaction of intervention phase × side did not have a significant effect. However, as expected, there was a significant two-way interaction for the intervention phase × treatment group [F(2,174) = 41.3; *p* < 0.001].”

**Changes to Section 3.3. Baseline Phase to the Final Resting Phase**

The first sentence should be corrected to: “For percentage changes in SC, as shown in Table 4, the 3 × 2 mixed-measures ANOVA revealed a significant group × site interaction [F(3,174) = 16.4; *p* < 0.001].”

The authors state that the scientific conclusions are unaffected. This correction was approved by the Academic Editor. The original publication has also been updated.

## Figures and Tables

**Table 1:** Baseline values of sex, age, weight, height, BMI, and room-temperature measurements of the three groups. Values are expressed as mean ± SD.

Variable	Slider Technique Group (n ^a^ = 30)	Tensioner Technique Group (n = 30)	Control Group (n = 30)	*p*-Value
Sex (Male/Female)	53%/47%	53%/47%	47%/53%	-
Age (Years)	21.70 ± 2.63	26.30 ± 3.17	27.00 ± 3.16	0.11
Weight (kg ^b^)	73.20 ± 7.92	73.10 ± 7.97	73.80 ± 7.95	0.29
Height (cm ^c^)	173.80 ± 1.79	173.10 ± 1.78	174.40 ± 1.79	0.59
BMI ^d^ (kg/cm^2 e^)	23.70 ± 2.51	23.80 ± 2.54	24.00 ± 2.53	0.35
Room temperature (°C ^f^)	24.70 ± 2.51	24.60 ± 2.49	24.60 ± 2.51	0.51

^a^ n: number of partitions; ^b^ kg: kilogram; ^c^ cm: centimeter; ^d^ BMI: body-mass-index; ^e^ kg/cm: kilogram force per square centimeter; ^f^ °C: degrees Celsius.

**Table 3:** Outcome-measure values from the intervention phase to the final resting phase for the slider, tensioner, and control groups for the left and right legs. Values are presented as mean ± SD.

Side	Outcome Measures	Slider Group	Tensioner Group	Control Group	*p*-Value
Left Leg	Percentage change in skin conductance (μMhos ^a^)	−34.21 ± 23.77	−39.18 ± 20.08	−10.57 ± 14.14 ^bc^	<0.001
Right Leg	Percentage change in skin conductance (μMhos ^a^)	−38.62 ± 22.53	−26.58 ± 27.56	−10.02 ± 15.65 ^bc^	<0.001
Percentage change in Systolic Blood Pressure (mmHg ^d^)		4.55 ± 0.11	4.55 ± 0.11	4.55 ± 0.11	0.95
Percentage change in Diastolic Blood Pressure (mmHg ^d^)		4.65 ± 0.14	4.65 ± 0.14	4.65 ± 0.14	0.06
Percentage change in Body Temperature (°C ^e^)		36.92 ± 0.39	36.99 ± 0.36	36.81 ± 0.43 ^bc^	<0.001

^a^ μMhos: mean anticipatory and task-activity skin conductance level; ^b^ denotes significant difference compared to slider group; ^c^ denotes significant difference compared to control group; d mmHg: millimeters of mercury; ^e^ °C: degrees Celsius.
