# Supplementary material for: Comparative Effects of Neurodynamic Slider and Tensioner Mobilization Techniques on Sympathetic Nervous System Function: A Randomized Controlled Trial
Source: J Clin Med. 2024 Aug 28;13(17):5098. doi: 10.3390/jcm13175098 (PMC11396284; doi:10.3390/jcm13175098)
Supplement: Supplementary file 1 [file jcm-13-05098-s001.zip › jcm-3170837-supplementary.docx]

**Appendix S**

1. **Standardization of the Initial Setup**
2. **Participant Preparation**
3. **Standardization of participants in the sympathetic slump position**
4. **Final Equipment Check:**
5. **Standardization of the intervention phase**
6. **Standardization of the three intervention groups**

Before the start of testing, each participant received written instructions about the aim and procedures of the study, followed by signing the informed consent form.

**A. Standardization of the Initial Setup**

1. **Participant Allocation and Initial Setup:**

- The therapist asks each participant for their reference number **before** they enter the practical room.
- The therapist checks the list to see which group the participant belongs to.
  - The therapist **DOES NOT TELL** the group allocation to the participant and the data collector!

**2. Pre-Experiment Data Collection:**

- The therapist records the participant’s weight, height, and room temperature and double-checks their personal information.
- The therapist fills out the pre-experimental information sheet before each set of recordings.

**B. Participant Preparation**

1. **Initial Equipment Standardization:**
   - The therapist begins the standardization process as the participant is positioned on the plinth.
   - The therapist positions the thermo-camera © at the correct height, ensuring it is aimed to capture the participant’s entire body. The therapist then asks the data collector to check the monitor.
   - The data Collector gives the following instructions to the therapist: “Respond with “Yes, the camera is ok” or “No, we have to adjust it”. If adjustments are needed, the data collector guides the therapist without revealing any values seen on the screen.
2. **Blood Pressure Cuff Placement:**
   - The therapist places the cuff of the blood pressure monitor on the participant’s left arm, ensuring it is secure and positioned correctly.
3. **Skin Electrode Preparation:**
   - The therapist cleans the ventral surfaces of the 2^nd^ and 3^rd^ toes on both of the participant’s feet using an isopropyl alcohol tissue.
   - The therapist allows the cleaned area to dry for 10 seconds to ensure the removal of any sweat residue or unwanted skin that could interfere with data readings.
4. **Electrode Placement:**
   - The therapist attaches the disposable electrodes on the 2^nd^ and 3^rd^ toes of each foot.
   - The therapist stabilizes the pads on the participant’s toes using sports tape to maintain optimal electrode contact.
5. **Avoiding Circulation Restriction:**
   - The therapist ensures that the stabilization tape on the participant’s toes is not over-tightened to prevent circulation restriction. The therapist asks the participant if they feel any discomfort.
   - The therapist also performs a capillary refill test to assess circulation in the area.
6. **Final Participant Positioning:**
   - Once the electrodes are correctly positioned and secured, the therapist instructs the participant to assume a long sitting position on the plinth.

**C. Standardisation of participants in the sympathetic slump position**

**1. Initial Positioning:**

- The therapist requests the participant to sit in the long sitting position on the plinth, with legs fully extended and knees straight.
- The therapist ensures that the participant’s spine is straight, with their head in line with the body, facing forward.
- The therapist instructs the participant to rest their hands on the sides in a neutral position.

**2. Adjusting Plinth Height:**

- The therapist adjusts the height of the plinth to an optimal level that allows for better control during the neurodynamic maneuvers, taking into consideration the participant’s height. (Check the hip point of the therapist.)

**3. Securing the Sacrum and Knees:**

- The therapist applies a sacral block to stabilize the participant’s sacrum.
- The therapist applies a fixation belt to secure the participant’s knees in the extended position on the plinth.

**4. Positioning the Hands:**

- The therapist instructs the participant to clasp their hands together behind their body.

**5. Flexing the Spine:**

- The therapist instructs the participant to flex their head forward first, followed by flexion of the thoracic spine. Adjustments are needed based on the intervention group to which the participant belongs:
  - **Control Group:** Neutral position.
  - **Sliding Technique:** Less flexed and closer to neutral position
    - **Verbal Instruction:** “Gently lower your chin towards your chest, as if you are nodding ‘yes.’ We are aiming for a slight flexion in your neck. Imagine there is a small gap, that should remain between your chin and your chest. Keep your eyes looking slightly downwards and maintain this position without straining or feeling discomfort.”
  - **Tensioner Technique:** The therapist ensures that the participant reaches the maximum range of cervical and thoracic flexion, using the ‘wind up’ feeling as a guide to identify the correct range.
    - **Stabilizing the Head and Foot:**
- The therapist stabilizes the participant’s head with one hand.
- The therapist uses the other hand to hold the lateral aspect of the participant’s intervention foot (with the therapist’s grip able to move the foot in dorsiflexion).

**7. Final Instructions Before the Experiment:**

- The therapist instructs the participant to remain relaxed during the entire experimental process.
- During the experiment, the therapist asks the participant not to sleep, cough, speak, or sneeze.
- The therapist repeats to the participant that the experiment will proceed through three phases, and the participant will be guided on what to do at the beginning of each phase by the data collector.
- Each participant must pass through three phases to complete the experiment. The phases include the Pre-Intervention Phase, where initial readings and baseline recordings are taken; the Intervention Phase, where the neurodynamic techniques are applied with rest periods in between; and the Post-Intervention Phase, where final readings are recorded. This structured approach ensures consistency and accuracy across the 20-minute total experiment period (information included in the participant information sheet).
- The therapist instructs the participant not to respond verbally or make any movements or sounds at any point during the experiment.

**D. Final Equipment Check:**

- After the participant is in the final position, the therapist asks the data collector to ensure the readings from the thermo-camera©, ambulatory blood pressure monitor, and Biopac acquisition software (skin conductance activity) are collected correctly and accurately.

**E. Standardisation of the intervention phase**

1. **Initial Stabilization and Baseline Recording:**
   - The therapist starts the experiment with the 8-minute stabilization phase to achieve a physiological resting state.
   - The therapist gives the following instructions to the data collector: “Please check the monitor”. When the line is stable, the data collector asks the participant to take a deep breath.
     - **Data collector’s instructions to participant:** “Please take a deep breath through your nose, slowly and deeply, and then exhale gently.”
     - The data collection continues with a 2-minute baseline recording period, during which the SC is continuously recorded using the Biopac equipment.
     - The data collector collects the baseline body temperature and blood pressure using the blood pressure monitor and the thermo-camera ©.
2. **Intervention Phases:**
   - The therapist conducts three 1-minute intervention phases on the ‘intervention leg,’ each separated by a 1-minute rest period based on the group allocation. (The data collector checks and indicates the timer for identifying the 1-minute!!)
   - Refer to the ‘Standardization of the Three Intervention Groups’ for further instructions.
3. **Guidance During the Experiment:**
   - The therapist listens for the data collector’s verbal cue ‘Intervention Phase 1, 2, or 3’ to start each intervention phase.
   - The therapist maintains the correct procedure while the data collector places markers on the Biopac graph to indicate the start and end of each intervention phase.
4. **Post-Intervention Data Collection:**
   - Upon completion of the intervention phases, the therapist initiates the 5-minute resting period.
   - The data collector checks and indicates the time for identifying the 5-minute!!)
   - The data collector records the post-intervention body temperature and blood pressure using the blood pressure monitor and the thermo-camera ©.
5. **Cleanup:**
   - The therapist removes the electrodes from the participant’s toes and cleans the area with isopropyl alcohol wipes.
   - The therapist removes the blood pressure cuff from the participant.

**F. Standardisation of the three intervention groups:**

- **Control Group:**
  - **Initial Positioning:** The therapist ensures that the participant comfortably maintains the Sympathetic Slump Position as previously described.
  - **Monitoring:** The therapist monitors the participant so that to maintain this position for the total duration of 20 minutes without performing any specific maneuvers or interventions.
  - **Posture Check:** The therapist regularly checks the participant’s posture to ensure they maintain the correct position throughout the 20-minute period. (Every 5 minutes, check the timer.)
- **Sliding Technique group:**
  - **Initial Positioning:** The therapist ensures that the participant comfortably holds the Sympathetic Slump Position as previously described.
  - **Head Positioning:** The therapist maintains the participant’s head in a less flexed position, keeping it closer to neutral (chin and Flexion 1, 2 as practiced).
  - **Foot Dorsiflexion:** The therapist carefully performs full dorsiflexion of the participant’s foot. The movements should be smooth and controlled, focusing on allowing the nerve to glide through its pathway without creating significant tension.
  - **Dynamic Application:** The therapist applies the sliding technique dynamically, checking the timer as practiced. The therapist ensures that each sliding phase is followed by a relaxation interval (checking the timer as practiced).
  - **Observation and Adjustment:** The therapist continuously monitors the participant’s posture and the quality of the movement.
  - **Completion:** The therapist ensures that the participant remains comfortable, and that the sliding technique is performed consistently and effectively throughout the session.
- **Tensioner Technique Group:**
  - **Initial Positioning:** The therapist ensures that the participant comfortably holds the Sympathetic Slump Position as previously described.
  - **Head and Foot Positioning:** The therapist fully flexes the participant’s cervical spine forward, ensuring the head is fully bent forward. Simultaneously, the therapist performs full dorsiflexion of the participant’s intervention foot, enhancing neural tension throughout the nerve pathway.
  - **Dynamic Application:** The therapist applies the tensioner technique dynamically, alternating between the fully tensioned position and a relaxed state. The therapist checks the timer as practiced, ensuring a relaxation interval follows each tension phase.
  - **Movement Execution:** The therapist repeats the simultaneous full flexion of the cervical spine and dorsiflexion of the foot, maintaining a controlled and steady pace. The therapist ensures that the participant’s position and the technique are performed consistently during each cycle.
  - **Observation and Adjustment:** The therapist monitors the participant’s response throughout the procedure
  - **Completion:** The therapist ensures that the participant remains comfortable and that the tensioner technique is performed consistently and effectively throughout the session.
